# “Special needs” is an ineffective euphemism

**DOI:** 10.1186/s41235-016-0025-4

**Published:** 2016-12-19

**Authors:** Morton Ann Gernsbacher, Adam R. Raimond, M. Theresa Balinghasay, Jilana S. Boston

**Affiliations:** 1 University of Wisconsin-Madison, Madison, WI USA; 2 University of Kansas, Lawrence, KS USA

**Keywords:** Down Syndrome, Intellectual Disability, Free Association, Personal Connection, Psychiatric Disability

## Abstract

Although euphemisms are intended to put a more positive spin on the words they replace, some euphemisms are ineffective. Our study examined the effectiveness of a popular euphemism for persons with disabilities, *special needs*. Most style guides prescribe against using the euphemism *special needs* and recommend instead using the non-euphemized term *disability*; disability advocates argue adamantly against the euphemism *special needs*, which they find offensive. In contrast, many parents of children with disabilities prefer to use *special needs* rather than *disability*. But no empirical study has examined whether *special needs* is more or less positive than the term it replaces. Therefore, we gathered a sample of adult participants from the general population (*N* = 530) and created a set of vignettes that allowed us to measure how positively children, college students, and middle-age adults are viewed when they are described as having *special needs*, having *a disability*, having a certain disability (e.g*.*, *is blind*, *has Down syndrome*), or with no label at all. We predicted and observed that persons are viewed more negatively when described as having *special needs* than when described as having *a disability* or having a certain disability, indicating that *special needs* is an ineffective euphemism. Even for members of the general population who have a personal connection to disability (e.g., as parents of children with disabilities), the euphemism *special needs* is no more effective than the non-euphemized term *disability*. We also collected free associations to the terms *special needs* and *disability* and found that *special needs* is associated with more negativity; *special needs* conjures up more associations with developmental disabilities (such as intellectual disability) whereas *disability* is associated with a more inclusive set of disabilities; and *special needs* evokes more unanswered questions. These findings recommend against using the euphemism *special needs*.

## Significance

Our research question grew directly from a real-world problem. As the examples quoted in this study demonstrate, the euphemism *special needs* occurs in everyday vernacular as well as in the scholarly literature. Thus, empirically examining the effectiveness of the euphemism *special needs* is use-inspired basic research.

## Background

Euphemisms are “expressions used in place of words or phrases that otherwise might be considered harsh or unpleasant” (Annan-Prah, 2015). Employees are not fired; they are *let go*. Animals are not euthanized; they are *put to sleep*. Humans don’t die; they *pass away* (Crespo-Fernández, 2006). In years past, pregnant women were referred to as *being in a family way*, and children entering puberty were referred to as *going around the corner* (Riggs, 2013). Euphemisms occlude uncomfortable topics.

The word *euphemism* derives from the Greek word *eupheme*, which is the act of using language to effect a good omen (in contrast to *blaspheme*; Burkhardt, 2010). When Portuguese sailors were forced to navigate The Cape of Storms, they renamed the rocky headland *The Cape of Good Hope*; when ancient Romans conquered the city of Maleventum (which means bad result), they changed the city’s name to *Beneventum* (now called Benevento; Griffin, 1985). Euphemisms, words used to effect good omens, have been popular for millennia.

In modern day, euphemisms are often used intentionally to deceive or manipulate others. Rather than speaking of economic recessions, politicians speak of *minus growth*; rather than speaking of military attacks, they speak of *missions* (Burkhardt, 2010); rather than bribes, they speak of *soft commissions* (Rittenberg, Gladney, & Stephenson, 2016). Such Orwellian double-speak lives not only in the lexicons of politicians, but also the everyman. If we cannot afford to buy a new car, we buy a *pre-owned* car rather than a used car; if we cannot afford to fly first class, we fly *coach* rather than second class (Burkhardt, 2010).

Euphemisms are believed to serve both those who produce them (speakers and writers) and those who receive them (listeners and readers). Linfoot-Ham (2005, p. 228) believes that euphemisms “protect the speaker/writer, hearer/reader, or all of the above.” Bowers and Pleydell-Pearce (2011, p. 2) believe that euphemisms “allow speakers (and listeners) to think about issues that might otherwise be avoided.” Allan and Burridge (1991, p. 11) believe that euphemisms “avoid possible loss of face: either one’s own face or … that of the audience.” However, laboratory studies demonstrate that euphemisms are more likely to be produced in service of saving the producer’s face rather than the recipient’s face (McGlone & Batchelor, 2003).

Although the explicit purpose of euphemisms is to avoid offense, some euphemisms cause offense. Doctors can be offended when they are called *providers* (Sergel, 2015). Church members can be offended when they are called *customers* (La Cour & Kromann, 2011). Patients can be offended when their experience with cancer is euphemized as a *journey* (Appleton & Flynn, 2014), and workers can be offended when their employment termination is conveyed as *downsizing* (Vickers, 2002). The very words intended to sugar coat can be more distasteful than the words the euphemisms displace.

Euphemisms can also be ineffective. The doctor-preferred euphemism *you have fluid on your lungs because your heart is not pumping hard enough* is no more effective to patients at risk of heart failure than the non-euphemized term *heart failure* (Tayler & Ogden, 2005). The nurse-preferred euphemism *your weight may be damaging your health* is demonstrably less effective to patients who are obese than the non-euphemized term *obese* (Swift, Choi, Puhl, & Glazebrook, 2013; Tailor & Ogden, 2009). The goal of our study was to examine the effectiveness of a popular euphemism for persons who have disabilities.

## *Special needs* as a euphemism for disability

Euphemisms for disability are popular—so popular that style guides prescribe against using euphemisms for persons who have disabilities. For example, the American Psychological Association (2010, p. 76) tells writers to “avoid euphemisms” for disability, such as “*special*, *physically challenged*, *handi-capable*” because those euphemisms “are condescending.” Similarly, “bypass condescending euphemisms” is a primary recommendation of the Research and Training Center on Independent Living (2013), who note that “terms such as *special*, *handi-capable*, *differently abled* and *challenged* reinforce the idea that people cannot deal honestly with their disabilities.” The American Speech-Language-Hearing Association also prescribes against using euphemisms for disability because euphemisms cannot “hide disability, but they can produce confusion” (Folkins, 1992).

**Fig. 1:**
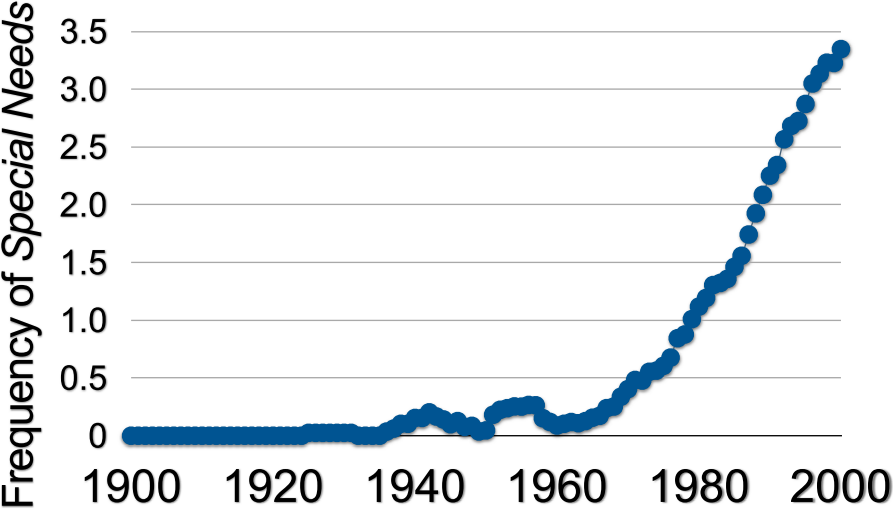
Percentage (10^−6^) of published books (from 1900 to 2000) in which the term *special needs* appears, according to Google NGram

Over the past few decades, the term *special needs* has become a popular euphemism for disability (Berger, 2013). Rather than identifying a person as *having a disability* or having a certain disability (e.g., *Anika is blind*, *Bruce has ADHD*), the person is euphemized as *having special needs*. Figure 1 demonstrates the steeply rising popularity of the term *special needs*, based on Google’s NGram count in published books dating back to 1900. Currently, Google Scholar indexes over a million scholarly articles with the term *special needs*, and Amazon.com sells nearly 5000 books with the euphemism *special needs* in their title. *Special needs* is an increasingly popular euphemism.

**Fig. 2:**
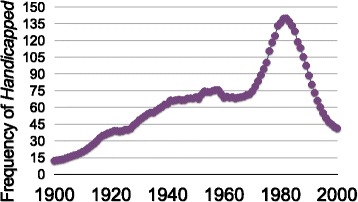
Percentage (10^−5^) of published books (from 1900 to 2000) in which the term *handicapped* appears, according to Google NGram

The origin of *special needs* as a disability euphemism is unclear. Guralnick (1994) reports in the 1990s changing the wording of a 1980s questionnaire for parents from *handicapped children* to *children with special needs*, suggesting that the euphemism had taken hold by the end of the 20th century. Figure 2 demonstrates the declining popularity of the term *handicapped* based on Google’s NGram. Shapiro-Lacks (2013) proposes that the euphemism *special needs* morphed from the term Special Olympics, established in the late 1960s, and the concept of special education, also established in the 1960s. “At some point, people with disabilities began to be referred to as *special*, our needs as *special needs*, and our demographic as *the special needs population*,” writes Shapiro-Lacks (2013).

However, *special needs* is not a legal term. In nearly a thousand pages of US law, including the Elementary and Secondary Education Act of 1965, the Rehabilitation Act of 1973, the Individuals with Disabilities Education Act of 1990, and the Americans with Disabilities Act of 1990 and 2014, the term *special needs* occurs only a dozen or so times. And never once are children with disabilities or adults with disabilities referred to as *children with special needs* or *adults with special needs*. Rather, individuals with disabilities are always referred to in US law as *individuals with disabilities*.

Federal laws use the term *special needs* only to refer to the distinctive requirements of various groups. For example, the 2004 Individuals with Disabilities Education Act tasks a federal research center to “examine the *special needs* of limited English proficient children with disabilities.” The 1984 amendment to the Vocational Education Act tasks a state board to “assess the *special needs* of groups of individuals,” and those groups include “individuals who are single parents or homemakers and individuals who participate in programs designed to eliminate sex bias and stereotyping in vocational education.” The 1974 amendment to the Elementary and Secondary Education Act tasks a state agency to assess the *special needs* of the Commissioner of Education (a federal position now called the Secretary of Education). Thus, federal laws do not use *special needs* as a euphemism for disability.

Most style guides also prescribe against using the euphemism *special needs*. For example, the National Center for Disability Journalism (2015, p. 23) warns that “the word *special* in relationship to those with disabilities is now widely considered offensive because it euphemistically stigmatizes” persons with disabilities. Therefore, the National Center for Disability Journalism (2015, p. 23) advises to “avoid using these terms [*special* and *special needs*]” and instead “cite the specific disability or disabilities in question.” Similarly, the Research and Training Center on Independent Living (2013) advises that “the use of *special needs* is offensive … Just say *individuals with disabilities*.”

Disability advocates argue adamantly against using the euphemism *special needs*. Kailes (2010) deems *special needs* “an offensive euphemism” that is “patronizing, inappropriate, and distancing.” Woodward (1991) and Linton (1998, p. 14) deem *special needs* a condescending euphemism, promoted by paternalistic “do-gooders.” Rucker (2014) deems *special needs* akin to other “socially unacceptable words” and “ethnic/social slurs no longer tolerated.” Indeed, in an international survey of English-speaking persons with disabilities conducted by the BBC, *special* ranks fourth in a list of terms considered offensive; *special* was barely beaten out by the slurs *spastic* and *retard* (Ouch!, 2003).

Several campaigns have lobbied to remove the euphemism *special needs*. Using social media, Lawrence Carter-Long (Carter-Long, 2016) advanced the Twitter hashtag #NotSpecial alongside the hashtags #SayTheWord and #Disabled and UK television star Josh Reeves (Reeves, 2015) launched the #Don’tCallMeSpecial campaign. In the Disability Studies literature, UK scholar Colin Barnes campaigned to replace *special education* with *inclusive education*, replace *special educational needs* with *unmet educational needs*, and replace the euphemism *children with special education needs* (*SEN*) with the non-euphemized term *disabled children* (Barnes & Sheldon, 2007).

However, parents of children with disabilities and professionals who work with children and adults with disabilities are not nearly so comfortable with the non-euphemized terms *disabled* and *disability* (Foundation for Jewish Summer Camps, 2015; Steinberg, 2013). As one mother relates, “I don’t like the term *special needs*, but … I don’t like the word *disabled* or *disability* any better” (I Am The Giraffe, 2010). Another mother relates, “Every single time I use that term [*special needs*], I flinch inside; it just sounds so…stiff. Still, it’s less harsh than the reality of *disabled*” (Love That Max: Special Needs Blog, 2008). One father relates, “I prefer the term *special needs* [because] I feel like it is a little bit less derogatory,” and another father relates, “*special needs* maybe sounds more positive, you know, than *disability*” (Sams, 2012, p. 147).

The goal of our study was to examine empirically whether the euphemism *special needs* is indeed more positive than the non-euphemized term *disability* among the general population, as well as among parents of children with disabilities, people who provide services to persons with disabilities, and other people with a personal connection to disability. We constructed a set of vignettes, which we manipulated to allow us to answer empirically the question of whether *special needs* is an effective euphemism for *disability*.

## Vignettes as a tool for studying attitudes

Vignettes, “short stories about hypothetical characters in specified circumstances” (Finch, 1987, p. 105), were pioneered in the 1960s as a tool to study attitudes (Goldberg, 1968; Nosanchuk, 1972). Since then, vignettes have been used to study a range of attitudes, most notably prejudicial attitudes, including sexism (Baron, Burgess, & Kao, 1991), racism (Gilbert, 1998; Haider et al., 2011), ethnic prejudice (Hudley & Graham, 2001; Mills & Gaia, 2012), religious prejudice (Pitner, Astor, Benbenishty, Haj-Yahia, & Zeira, 2003), homophobia (St. Lawrence, Husfeldt, Kelly, Hood, & Smith, 1990), xenophobia (Caprariello, Cuddy, & Fiske, 2009), and ableism (prejudice against disabled people; Butler & Gillis, 2011; Grewal, Joy, Lewis, Swales, & Woodfield, 2002; Matthews, Ly, & Goldberg, 2015; Nevill & White, 2011; Werner, 2015).

Vignettes are ideal for answering our research question because they allow subtle manipulation; in fact, the manipulation can be limited to only one or two words. For example, to examine whether racism affects hiring decisions, employers can be given identical descriptions of job applicants that differ only by the applicant’s name (e.g., *Jamal Jones* versus *Greg Jones*; Bertrand & Mullainathan, 2004). To examine whether sexism affects teaching evaluations, university students can be given identical descriptions of instructors that differ only by the instructor’s gender (Kierstead, D’Agostino, & Dill, 1988).

Via manipulation of only one or two words, vignettes have been used to examine a range of use-inspired research questions, including driving accident responsibility (Davies & Patel, 2005) and sexual assault culpability (Neal, 2015). Most relevant to our study, vignettes have been used to examine the effectiveness of euphemisms (e.g., referencing used cars as *pre-owned* or bribes as *soft commissions*; Gladney & Rittenberg, 2005; Rittenberg et al., 2016). However, to our knowledge, no study has examined the effectiveness of the euphemism *special needs*, which was our study’s goal.

## Overview of the current study

To examine empirically the effectiveness of the euphemism *special needs*, we constructed several vignettes. An example appears in Table 1. Each vignette described an envisioned situation in which participants were tasked with choosing a character. For example, in the vignette presented in Table 1, participants envisioned being a university freshman who has decided to live in the dormitories; therefore, the participants were tasked with choosing a roommate. For another vignette, participants envisioned being a second-grade teacher whose principal needed to place one more child in their already full-to-capacity class; therefore, participants were tasked with choosing a new second-grade student. As another example, participants envisioned being a middle-age employee who is told to complete an important project in collaboration with a co-worker; therefore, participants were tasked with choosing a workplace collaborator.

**Table 1:** Example vignette

You are a freshman about to enter your first year of college at a large state university. You decide to live in the dormitories and need to select a roommate. Based on the following descriptions, select your first, second, third, and fourth choice for the person you would like to have as a roommate.
Roommate A is a 19-year-old history major. Roommate A is from a small town, 40 minutes away. Roommate A likes to play soccer, and watch movies, with an extensive DVD collection that they are planning to bring to your dorm room. Roommate A prefers to study at the library. Roommate A is in a serious romantic relationship that started in high school and might have their significant other visiting in your room, even overnight.
Roommate B is an 18-year-old business major and has special needs (material set 1). Roommate B is an 18-year-old business major and has a disability (material set 2). Roommate B is an 18-year-old business major and is blind (material set 3). Roommate B is an 18-year-old business major (material sets 4, 5, and 6). Roommate B is from a small town, four hours away. Roommate B enjoys hiking, camping and going on long runs, and is a vegan. Roommate B studies mainly at the library. When in your room, Roommate B will be very social and keep your dorm room door open to everyone on your floor.
Roommate C is a 19-year-old Spanish major. Roommate C is from out of state. Roommate C likes being active, hanging out with friends, and practicing the guitar in your room. Roommate C is a morning person, going to sleep around 9:30 p.m. and waking up around 6:00 a.m. every day. Roommate C will use those early mornings to study. Roommate C is personable and loves to talk but can also be a bit intense.
Roommate D is an 18-year-old psychology major. Roommate D is from the largest city in the state. Roommate D likes to draw, play tennis, and listen to loud music. Roommate D always studies with music playing through their headphones. Roommate D is a night owl and, as an only child, has never shared a room before.

For each vignette, participants chose among four characters (e.g., roommate A, B, C, and D). Participants ranked their choices first to last. Using a within-subjects design, we manipulated the following three experimental conditions:

- **Has a disability**: The to-be-chosen character was described as having a disability, for instance, “Roommate B is an 18-year-old business major and has a disability.”
- **Has a certain disability**: The to-be-chosen character was described as having a certain disability, for instance, “Roommate B is an 18-year-old business major and is blind.”
- **Has special needs**: The to-be-chosen character was described as having special needs, for instance, “Roommate B is an 18-year-old business major and has special needs.”

We also manipulated a fourth, no-label control condition:

- **No label (control)**: The to-be-chosen character was not described with any label, for instance, “Roommate B is an 18-year-old business major.”

With these found conditions we were able to test the effectiveness of the euphemism *special needs* against the non-euphemized term *disability*. We were also able to test the effectiveness of the euphemism *special needs* against the label of a specific disability (e.g., *is blind*). And we were able to test the effectiveness of the euphemism *special needs* against no label at all in our control condition. If *special needs* is an ineffective euphemism, then characters described as having *special needs* should be more likely to be chosen last, which is what we predicted. Indeed, we predicted that characters described as having *special needs* would be chosen last even more often than characters described as having *a disability* and characters described as having a certain disability (e.g., *is blind*).

The to-be-chosen character was a child in a third of our vignettes, was a college student in a third of our vignettes, and was a middle-age adult in a third of our vignettes. Therefore, we could investigate whether *special needs* is an ineffective euphemism regardless of the age of the person being described.

After reading all the vignettes and ranking all the to-be-chosen characters, participants provided free associations to the terms *special needs* and *disability*. We predicted that participants would have more negative associations to the euphemism *special needs* than to the non-euphemized term *disability*. Although our sample of research participants was drawn from the general population of US adults, we collected demographic information that allowed us to examine our research questions with participants who have a close relationship to disability (e.g., as parent of a child with a disability or as a professional who works with persons with disabilities) compared with participants who do not have a close relationship to disability.

## Methods

### Materials

Six vignettes were written and are available at http://www.gernsbacherlab.org/, as well as http://dx.doi.org/10.17605/osf.io/nyv4s. For two of the six vignettes, the to-be-chosen character was a college student, either a dorm roommate for freshman year or a week-long cabin-mate for an alternative spring break. For another two of the six vignettes, the to-be-chosen character was a child, either a new student in a second-grade classroom or a new player for a youth-basketball team. For the remaining two vignettes, the to-be-chosen character was a middle-age adult, either a workplace collaborator or a cooking class partner.

For each of the six vignettes, descriptions of four characters were written. As Table 1 illustrates, the four descriptions stated several facts about each character. For example, for the freshman dorm roommate vignette, each description stated the roommate’s major (e.g., business; history; Spanish); the roommate’s hometown (e.g., from a small town, 40 minutes away; from out of state; from the largest city in the state); the roommate’s hobbies (e.g., likes to watch movies; enjoys hiking; likes hanging out with friends); where the roommate prefers to study (e.g., library; dorm room); and a one-sentence description of the roommate’s personality and behavior (e.g., is personable and loves to talk but can be a bit intense).

To avoid gender bias, none of the descriptions identified the to-be-chosen character’s gender. A series of pilot studies ensured that the four character descriptions for each of the six vignettes were roughly equivalent in preference prior to our adding information about the character’s special needs or disability.

Six material sets were formed, as illustrated in Appendix Table 2. Each of the six material sets contained each of the six vignettes, in the same order (e.g., the freshman roommate vignette preceded the new second-grade student vignette, which preceded the middle-age adult workplace collaborator vignette, and so forth in all six material sets). To occlude the purpose of the study, only three of the six vignettes in each material set were assigned to the three experimental conditions; the other three vignettes in each material set were assigned to the fourth, no-label control condition.

Six different disabilities were used for the certain disabilities. Each of those six disabilities was assigned to only one to-be-chosen character and only one vignette: *is blind* (freshman roommate B), *has epilepsy* (workplace collaborator C); *is autistic* (new basketball team player D); *has Down syndrome* (new second-grade student B); *has ADHD* (spring break cabin-mate C), and *is deaf* (cooking class partner D).

Therefore, in each of the six material sets, only one of the 24 to-be-chosen characters was described as having a certain disability (e.g., *is blind*). Similarly, in each of the six material sets, only one of the 24 to-be-chosen characters was described as having *a disability*, and only one was described as having *special needs*. By applying our experimental manipulation to only three of the 24 to-be-chosen characters, we could better occlude the purpose of the study.

### Procedure

Participants were recruited to the study naïve to the study’s purpose. The study was titled, “Person Judgment Study,” and participants were told that they would participate in “a study in which you will read scenarios and rank preferences for people.”

Participants read each of the six scenarios; following each scenario, participants chose among the four characters, ranking their choices first to last. After reading all the vignettes and ranking all the to-be-chosen characters, participants completed a brief questionnaire soliciting the following demographic information: age; gender identity; where participants had lived the majority of the past five years; whether participants were native speakers of English; whether participants were university or college students, parents of elementary school age children, teachers of elementary school age children, middle-age adults, or middle-age adults with work responsibilities that include supervising other people (life roles captured in the vignettes); and whether participants were persons with a disability, parents of a child with a disability, relatives of a person with a disability, co-workers of a person with a disability, or good friends with a person with a disability (demographic information that allowed us to distinguish between participants with versus without a personal connection to disability).

It is important to stress that participants’ demographic information was not collected until after the participants completed reading all the vignettes and ranking all the to-be-chosen characters from first to last. Thus, the participants had no reason to believe that the purpose of the vignette task was related to disability.

Finally, the participants responded to two open-ended statements: “When I hear the term ‘special needs,’ the first few thoughts that come to my mind are ____” and then “When I hear the term ‘has a disability’ or ‘has disabilities,’ the first few thoughts that come to my mind are ____.” Participants were provided with five places to provide up to five free associations for each of the two statements. Again, it is important to stress that the participants’ free associations were not collected until after the participants completed reading all the vignettes and ranking all the to-be-chosen characters.

### Participants

The participants were 530 adults recruited through Amazon Mechanical Turk and compensated $2 for their participation in the 20-minute study. All participants were required to have a 93% or greater approval rating from their previous work on Mechanical Turk.

Participants were randomly assigned to one of the six material sets. Data from three participants were excluded because the participants responded too methodically (i.e., ranking the character presented first as their first choice, the character presented second as their second choice, the character presented third as their third choice, and so forth). Each of the remaining 527 participants had resided in the USA for the majority of the past five years and were native speakers of English.

The participants’ mean age was 30.2 years (standard deviation (SD) = 8.9 years; range = 18–69 years). The participants randomly assigned to the six material sets did not differ in age (*F*(5,521) = 0.52, *p* = 0.761). Of the 527 participants, 66% identified as male gender, 33% identified as female gender, and 1% identified outside the gender binary. The participants assigned to the six material sets did not differ in identifying as male versus female gender (*χ* ^2^(5) = 0.828, *p* = 0.975).

Twenty-nine percent of the participants identified as college students; 25% identified as middle-age adults; 10% identified as parents of elementary-school children; 9% identified as middle-age adults whose work responsibilities include supervising subordinates; 2% identified as teachers of elementary school children; and 38% identified as having none of these life roles. These percentages sum to greater than 100% because some participants identified as having more than one of these life roles. Participants randomly assigned to the six material sets did not differ in having these life roles (*χ* ^2^(5) = 8.828, *p* = 0.116; *χ* ^2^(5) = 2.720, *p* = 0.743; *χ* ^2^(5) = 1.904, *p* = 0.862; *χ* ^2^(5) = 4.598, *p* = 0.467; *χ* ^2^(5) = 6.758, *p* = 0.239, respectively) or as having none of these life roles (*χ* ^2^(5) = 4.561, *p* = 0.472).

Thirty-seven percent of the participants identified as having one or more personal connections to disability: 18% identified as a relative of a person with a disability; 16% identified as a good friend of a person with a disability; 6% identified as a person with a disability; 5% identified as a co-worker of a person with a disability; 4% identified as working with children who have disabilities; 4% identified as working with adults who have disabilities; and 3% identified as a parent of a child with a disability. These percentages sum to greater than 37% because some participants identified as having more than one personal connection to disability.

The majority of the participants, 61%, identified as not having any personal connection to disability, and 2% did not wish to identify whether they had a personal connection to disability. Participants randomly assigned to the six material sets did not differ in their personal connection to disability (*χ* ^2^(5) = 5.430, *p* = 0.365).

## Results

### Characters’ frequency of being chosen last

**Fig. 3:**
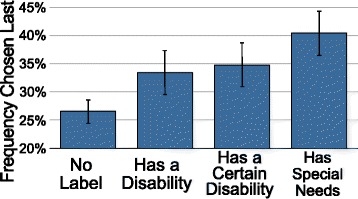
Frequency with which characters were chosen last as a function of four experimental manipulations: *No Label*, the characters were serving as a control and therefore no disability label was included in the characters’ description; *Has a Disability*, the characters were described as having *a disability*; *Has a Certain Disability*, the characters were described as having a specific disability (e.g., “is blind”); and *Has Special Needs*, the characters were described as having *special needs*. Error bars are ±95% confidence intervals

Figure 3 presents the frequencies with which characters were chosen last as a function of the four experimental manipulations: “No Label”, the characters were serving as a control and therefore no disability label was included in the characters’ description (26.6 ± 2.2%, 95% confidence interval); “Has a Disability”, the characters were described as having *a disability* (33.4 ± 4.0%); “Has a Certain Disability”, the characters were described as having a specific disability (e.g., “is blind,” 34.7 ± 4.0%); and “Has Special Needs”, the characters were described as having *special needs* (40.4 ± 4.2%).

As Fig. 3 illustrates, a one-dimensional chi-square goodness-of-fit test for mutually exclusive categories indicated that the frequencies with which characters were chosen last in the four experimental conditions (No Label, Has a Disability, Has a Certain Disability, Has Special Needs) differed significantly from chance (*χ* ^2^(3) = 15.16, *p* = 0.002).

As predicted, characters were significantly more likely to be chosen last when they were described as having special needs than when they were described as having a disability (*z* = 2.362, *p* = 0.009, one-tailed). Similarly, and as predicted, characters were significantly more likely to be chosen last when they were described as having special needs than when they were described as having a certain disability (*z* = 1.908, *p* = 0.028, one-tailed).

Characters were also significantly more likely to be chosen last when they were described as having special needs than when no (disability) label was included (*z* = 6.008, *p* < 0.001, one-tailed). In contrast, characters were equally likely to be chosen last when they were described as having a disability as when they were described as having a certain disability (*z* = −0.455, *p* = 0.653, two-tailed).

Thus, characters described as having special needs were most frequently chosen last, indicating that *special needs* is an ineffective euphemism. Characters described as having special needs were chosen last even more frequently than characters described as having a disability or a certain disability, suggesting that the euphemism *special needs* conveys more negativity than the non-euphemized term *disability* or the names of specific disabilities (e.g., *blind*). If *special needs* was an effective euphemism, then we would have observed that characters described as having special needs were chosen last less often than characters described as having a disability or a certain disability. But, as predicted, we observed just the opposite.

The pattern of results illustrated in Fig. 3 was obtained regardless of whether the to-be-selected character was a child, college student, or middle-age adult (*χ* ^2^(6) = 6.22, *p* = 0.399). Thus, *special needs* is an ineffective euphemism regardless of the age of the person to whom it is applied.

**Fig. 4:**
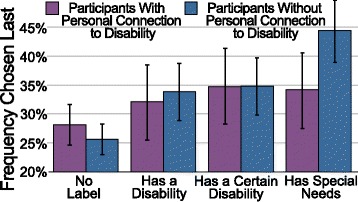
Frequency with which characters were chosen last as a function of four experimental manipulations (*No Label*, *Has a Disability*, *Has a Certain Disability*, and *Has Special Needs*) and whether participants have a personal connection to disability (e.g., is a relative, good friend, or co-worker of a person with a disability; works with children or adults who have disabilities; is a parent of a child with a disability; or is a person with a disability). Error bars are ±95% confidence intervals

However, as illustrated in Fig. 4, one aspect of the pattern of results was affected by whether the participants had a personal connection to disability. Participants with a personal connection to disability (37% of the sample) were less likely than participants without a personal connection to disability (61% of the sample) to choose characters last when the characters were described as having special needs (*z* = −2.284, *p* = 0.023, two-tailed). For the other three conditions, participants with a personal connection to disability resembled participants without a personal connection to disability (*z* = −0.014, *p* = 0.992, two-tailed, for “Has a Certain Disability”; *z* = −0.403, *p* = 0.689, two-tailed, for “Has a Disability”; and *z* = −0.616, *p* = 0.535, two-tailed, for “No Label”).

As Fig. 4 illustrates, participants with a personal connection to disability were equally likely to choose characters last when the characters were described as having special needs as when the characters were described as having a disability (*z* = 0.433, *p* = 0.667, two-tailed) or a certain disability (*z* = −0.108, *p* = 0.912, two-tailed). Thus, even for participants with a personal connection to disability, the euphemism *special needs* conveys as much negativity as the non-euphemized term *disability* or the names of specific disabilities (e.g., *blind*), again indicating that *special needs* is an ineffective euphemism.

### Participants’ associations to the terms *disability* and *special needs*

Participants’ associations to the prompts, “When I hear the term *special needs*, the first few thoughts that come to my mind are ____” and “When I hear the term *has a disability* or *has disabilities*, the first few thoughts that come to my mind are ____,” were coded by two of the co-authors, naïve to the participants’ demographics. The coders agreed on 96.83% of the codes and the disagreements were resolved by consensus.

Participants had been given five slots into which to write their associations to each of the two prompts (the term *special needs* and the term *has a disability* or *has disabilities*, which hereafter will be referred to as *disability*). On average, participants provided the same number of associations to *special needs* (*M* = 4.918; SD = 0.522) as to *disability* (*M* = 4.934; SD = 0.442, *t*(526) = 1.571, *p* = 0.117).

Participants’ associations were classified into ten mutually exclusive coding categories. Three of the ten coding categories captured affective associations: positive (e.g., “strong,” “capable,” or “acceptance”), negative (e.g., “annoying,” “helpless,” or “needy”), and neutral (e.g., “different,” “lifelong,” or “born that way”). Approximately 40% of participants’ associations were coded as belonging to one these three affective categories.

Four of the ten coding categories captured four types of disability: physical disabilities (e.g., “paraplegic,” “wheelchair user,” or “amputee”); developmental disabilities (“intellectual disability,” “autism,” or “Down syndrome”); sensory disabilities (e.g., “blind,” “deaf,” or “can’t talk”), and psychiatric disabilities (e.g., “mental illness,” “bipolar,” or “depression”). Approximately 30% of the participants’ associations were coded as belonging to one of these four types of disabilities.

The remaining three of the ten coding categories were empathy (e.g., “I want to help”), accommodations (“wheelchair,” “special education”), and requests for more information (e.g., “what’s wrong?”). Approximately 25% of the participants’ associations were coded as belonging to one of these three categories. The remaining associations, which composed less than 5% of the associations, were considered un-categorizable.

**Fig. 5:**
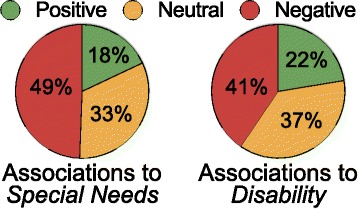
Relative proportion of participants’ associations that expressed affective sentiments that were *Positive*, *Neutral*, and *Negative* as a function of whether the participants were providing associations to the euphemism *special needs* or the term *disability*

Figure 5 presents the relative proportion of participants’ associations that expressed affective sentiments (positive, neutral, and negative) as a function of whether the participants were providing associations to the euphemism *special needs* or the term *disability*. As Fig. 5 illustrates, participants’ affective associations to *special needs* differed significantly from their affective associations to *disability* (*χ* ^2^(2) = 17.1, *p* < 0.001).

Participants produced significantly more negative associations to the euphemism *special needs* than to the term *disability* (*χ* ^2^(1) = 16.6, *p* < 0.001). Participants also produced significantly fewer positive associations and significantly fewer neutral associations to *special needs* than to *disability* (*χ* ^2^(1) = 6.12, *p* = 0.013 and *χ* ^2^(1) = 4.70, *p* = 0.030, respectively).

Participants produced significantly more negative and fewer positive associations to the euphemism *special needs* than to the term *disability*, regardless of whether they had a personal association to disability. Across the board, participants with a personal connection to disability provided fewer negative associations and more positive associations than did participants without a personal connection to disability (*χ* ^2^(2) = 55.8, *p* < 0.001). Nonetheless, both participants with and without a personal connection to disability produced more negative associations to *special needs* (41 and 54% of the participants’ affective associations, respectively) than to *disability* (33 and 45%, respectively). Thus, participants’ personal connection to disability did not diminish their negativity to the euphemism *special needs* (*χ* ^2^(1) = 0.012, *p* = 0.912).

Similarly, both participants with and without a personal connection to disability produced fewer positive associations to *special needs* (25 and 14% of the participants’ affective associations, respectively) than to *disability* (32 and 17%, respectively). Thus, participants’ personal connection to disability did not increase their positivity to the euphemism *special needs* (*χ* ^2^(1) = 0.059, *p* = 0.808).

**Fig. 6:**
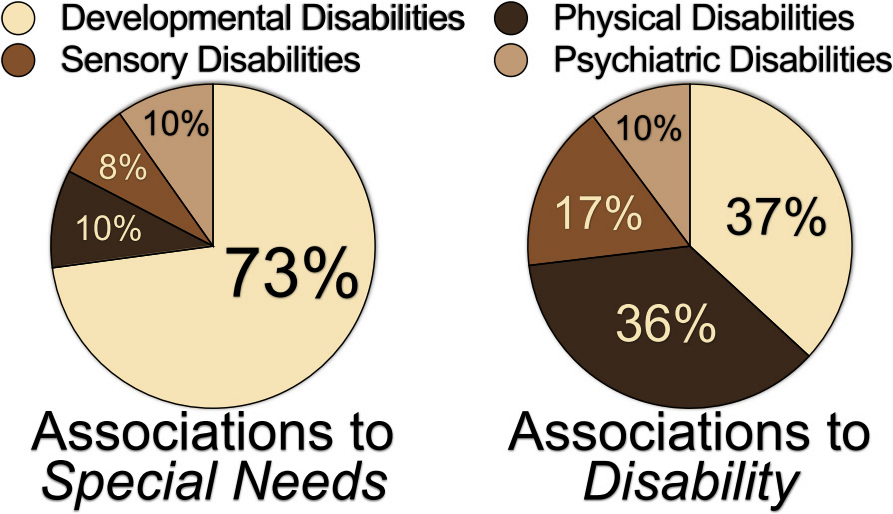
Relative proportion of participants’ associations that conveyed a type of disability, *Physical Disabilities*, *Developmental Disabilities*, *Sensory Disabilities*, and *Psychiatric Disabilities*, as a function of whether the participants were providing associations to the euphemism *special needs* or the term *disability*

Figure 6 presents the relative proportion of participants’ associations that conveyed a type of disability (developmental disabilities, physical disabilities, sensory disabilities, and psychiatric disabilities) as a function of whether the participants were providing associations to the euphemism *special needs* or the term *disability*. As Fig. 6 illustrates, participants associated significantly different disabilities with *special needs* than with *disability* (*χ* ^2^(3) = 233, *p* < 0.001).

Participants were significantly more likely to associate developmental disabilities with the euphemism *special needs* than with the term *disability* (*χ* ^2^(1) = 202, *p* < 0.001). Participants were significantly less likely to associate physical disabilities and sensory disabilities with *special needs* than with *disability* (*χ* ^2^(1) = 150, *p* < 0.001 and *χ* ^2^(1) = 29.6, *p* < 0.001, respectively). Participants were equally likely to associate psychiatric disabilities with *special needs* as with *disability* (*χ* ^2^(1) = 0.079, *p* = 0.779).

Lastly, participants were less likely to associate “Requests for more information” with the *special needs* (11%) than *disability* (18%; *χ* ^2^(1) = 12.1, *p* < 0.001). Participants were equally likely to associate “Empathy” with *special needs* (25%) and *disability* (28%; *χ* ^2^(1) = 1.11, *p* = 0.291). Participants were more likely to associate “Accommodations” with *special needs* (64%) than *disability* (55%; *χ* ^2^(1) = 11.8, *p* = 0.001). Many of the participants’ accommodations-related associations to *special needs* were related to special education or to “special” organizations and activities (e.g., Special Olympics).

## Discussion

The goal of our study was to examine empirically the effectiveness of the euphemism *special needs*.

We constructed a set of vignettes which allowed us to gauge how people who are described as having *special needs* are viewed in relation to people who are described as having *a disability*. If *special needs* is an ineffective euphemism, as we predicted, then people described as having *special needs* should be viewed more negatively than people described as having *a disability*, which is what we observed. Indeed, as we predicted, when people were described as having *special needs*, they were viewed more negatively than when they were described as having a specific disability (e.g*.*, *is blind*, *has Down syndrome*, *is autistic*, *has ADHD*).

We found that *special needs* is an ineffective euphemism regardless of whether the persons being described by the euphemism are children, college students, or middle-age adults. *Special needs* is an ineffective euphemism even in the eyes of people who have a personal connection to disability, such as parents of children with a disability or professionals who work with disabled children or adults. Even for people with a personal connection to disability, the euphemism *special needs* is no more effective than the non-euphemized term *disability*.

In our study, we also collected free associations to the terms *special needs* and *disability*. We found that *special needs* is associated with more negativity than *disability*; *special needs* conjures up more associations to developmental disabilities (such as intellectual disability, autism, or Down syndrome) whereas *disability* is associated with a broader, more inclusive set of disabilities; and *special needs* evokes more unanswered questions than *disability*. These associations again demonstrate that *special needs* is an ineffective euphemism. We propose that *special needs* is an ineffective euphemism because it is imprecise, it connotes segregation, and it implies special rights; *special needs* has become a dysphemism.

### *Special needs* is imprecise

Euphemisms are, by definition, vague expressions (Ramsay, 2002). But the euphemism *special needs* is so vague that it can be applied to a wide swath of individuals in various contexts. In the realm of child adoption, individuals with *special needs* can be children with a “range of conditions” that pose “a barrier to permanent adoption placement” (Tan, Marfo, & Dedrick, 2007, p. 1270), including being “minority and biracial” (Rosenthal & Groze, 1990, p. 476). In disaster preparation, individuals with *special needs* can be adults aged 50 years or older, adults who receive home health care, or adults who do not have access to a personal vehicle (Parsons & Fulmer, 2007).

In housing, individuals with *special needs* can be persons experiencing “homelessness, psychiatric illness, drug and alcohol rehabilitation, the elderly, HIV/AIDS, and women escaping domestic abuse” (Wilton, 2002, p. 318). In airline travel, they can be passengers with disabilities, passengers traveling with infants or animals, passengers traveling while pregnant, passengers requiring extra seating, passengers with peanut allergies, children traveling alone, and older adults (https://www.united.com/web/en-US/content/travel/specialneeds/default.aspx).

As Clapham and Smith (1990, p. 194) lament, “the precise groups considered to have *special needs* varies considerably and can include almost everyone.” For this reason, Kailes (2005, p. 3) argues that “continuing to use *special needs* does a disservice to every group included.” And “repeated pleas, over the years, from disability advocates to replace *special needs*, with more respectful, precise, segmented, and discrete groupings” should not “be ignored.”

### *Special needs* connotes segregation

We observed in our association data that the euphemism *special needs* evokes associations to special education, Special Olympics, and other special programs. Because these special programs mostly segregate persons with disabilities from persons without disabilities, *special needs* often connotes segregation (Woodward, 1991). Disability Studies scholars argue that *special needs* isolates persons with disabilities from the general population (Rucker, 2014) and creates distance between persons with versus without disabilities (Finkelstein & Stuart, 1996).

As Australian Senator Susan Boyce warns, “Anytime we allow people with a disability to be treated as *special* people who should live or learn or work or spend their leisure time in *special* places, we are shutting people with a disability out of the mainstream” (Australian Broadcasting Company 2007). A public demonstration of the connotation of *special needs* with segregation went viral a few years ago. At a family restaurant in Houston, one family asked to be re-seated farther away from another family which included a five-year-old son with Down syndrome. The father of the family who asked to be re-seated explained his motivation with the statement, “special needs kids should be kept in special places” (Broderick, 2013). Special places, special programs, special schools, and *special needs* connote segregation; in contrast, accessible places (rather than special places) and inclusive programs (rather than special programs) connote integration (Kailes, 2010).

### *Special needs* implies special rights

Construing disability as *special needs* enables misconstruing as *special rights* what are actually human rights, civil rights, or disability rights (Ballard, 1995). For example, a recent article in *The Chronicle of Higher Education* misconstrues legally mandated disability rights as *special rights*. The article erroneously describes Section 504 of the US Rehabilitation Act of 1973, which prohibits discrimination on the basis of disability in federally funded programs, as legislating “*special rights* and remedies for people with disabilities” (Rothstein, 2013). Section 504 does not legislate *special rights*; it legislates civil rights and federal protection from discrimination.

A more egregious misconstrual of basic rights as *special rights* was demonstrated in a viral complaint that a Canadian mother wrote to her neighbor (Daubs, 2013). The complaint centered around the neighbor’s disabled teenage grandson who plays joyously (and loudly) in his grandmother’s front yard. Among other offensive comments, the complaining mother wrote to her neighbor, “I hate people like you who believe, just because you have a special needs child, you are entitled to special treatment.” The “special treatment” was simply the disabled teenager’s basic right to play in his grandmother’s front yard. But referring to disabilities as *special needs* could have led to this teenager’s basic rights being misconstrued as *special rights* (see also Finkelstein & Stuart, 1996).

Like disability rights advocates, gay rights advocates have long argued that their rights should not be construed as *special rights*. The GLAAD (Gay & Lesbian Alliance Against Defamation 2016) Media Reference Guide identifies *special rights* as offensive and *equal rights* as preferred. As the Media Reference Guide explains, “Anti-gay extremists frequently characterize equal protection of the law for lesbian, gay, bisexual and transgender people as *special rights* to incite opposition” to basic, human, and civil rights. The negative implication of misconstruing human rights as *special rights* is unappreciated by many minority groups (Goldberg-Hiller & Milner, 2003; Marcosson, 2012; Rubin, 1998).

### *Special needs* has become a dysphemism

The data reported in this article suggest that *special needs* is so ineffective as a euphemism that it has become a dysphemism, particularly for members of the general population who do not have a personal connection to disability. Dysphemisms are terms that begin as euphemisms but become even more negative than the terms they were initially intended to euphemize (Gómez, 2009; Holder, 2002). Many contemporary slurs for members of racial, ethnic, or sexual minority groups began as euphemisms and became dysphemisms. Pinker (1994) deems such linguistic transition the euphemism treadmill.

Adams (1985, p. 44) explains that because euphemisms are “an effort to make something sound especially nice … a euphemistic formation can easily turn into its opposite.” The extreme effort to “prettify” a concept dramatizes not only the perceived negativity of the concept but also the perceived negativity of its euphemism.

It is unsurprising that *special needs* has become a dysphemism. Other disability terms have become not only dysphemisms but also dysphemistic metaphors (Pfaff, Gibbs, & Johnson, 1997). For example, among the definitions the *MacMillan Dictionary* (http://www.macmillandictionary.com/us) provides for the term *deaf* is the denotation “not willing to listen to something” (e.g., deaf to reason); for *blind*, one denotation is “unable to realize or admit the truth about something”; for *crippled*, one denotation is “damage[d] severely” or “prevent[ed] from working properly”; and for *lame*, “done without much effort in a way that seems as though you are not trying very hard.” Although *deaf*, *blind*, *crippled*, and *lame* originated as disability terms, they are now commonly used as dysphemistic metaphors.

The word *dumb* is now completely a dysphemistic metaphor. *Dumb* initially bore only a disability-specific denotation. It meant “permanently unable to speak.” But as the *MacMillan Dictionary* reports, the disability-related denotation of *dumb* is “now usually considered offensive,” and the preferred terminology for someone unable to speak is “speech impaired.” After *dumb* shed its disability-specific denotation of “permanently unable to speak,” it took on the disability-unrelated but still speech-related denotation of “temporarily unable or unwilling to speak, especially because you are very shocked” (e.g., *Some of the passengers were struck dumb with terror* or *I was struck dumb by the clerk’s rudeness*). The current and most popular denotation of *dumb* as “stupid” (e.g., *She’s so dumb she can’t even figure out her answering machine*) refers not at all to speech but reflects the (erroneous) connotation that persons with speech disabilities are intellectually inferior.

Our data suggest that *special needs* has already become a dysphemism (a euphemism more negative than the word it replaces). *Special needs* will likely become a slur, if it is not already, and it might eventually become a dysphemistic metaphor, akin to *dumb*, *lame*, *crippled*, *deaf*, and *blind*. These linguistic transitions, along with the data reported here, recommend against using the euphemism *special needs* and instead using the non-euphemized term *disability*.

## Appendix

**Table 2:** Experiment material sets

Material Set 1	Freshman Roommate A	New 2nd-Grade Student A	Workplace Collaborator A	Spring Break Cabin-Mate A	New Basketball Team Player A	Cooking Class Partner A
	**Freshman Roommate B … has special needs**	New 2nd-Grade Student B (control – no label)	Workplace Collaborator B	Spring Break Cabin-Mate B	New Basketball Team Player B	Cooking Class Partner B
	Freshman Roommate C	New 2nd-Grade Student C	**Workplace Collaborator C … has a disability**	Spring Break Cabin-Mate C (control – no label)	New Basketball Team Player C	Cooking Class Partner C
	Freshman Roommate D	New 2nd-Grade Student D	Workplace Collaborator D	Spring Break Cabin-Mate D	**New Basketball Team Player D … is autistic**	Cooking Class Partner D (control)
Material Set 2	Freshman Roommate A	New 2nd-Grade Student A	Workplace Collaborator A	Spring Break Cabin-mate A	New Basketball Team Player A	Cooking Class Partner A
	**Freshman Roommate B … has a disability**	New 2nd-Grade Student B (control)	Workplace Collaborator B	Spring Break Cabin-Mate B	New Basketball Team Player B	Cooking Class Partner B
	Freshman Roommate C	New 2nd-Grade Student C	**Workplace Collaborator C … has epilepsy**	Spring Break Cabin-Mate C (control)	New Basketball Team Player C	Cooking Class Partner C
	Freshman Roommate D	New 2nd-Grade Student D	Workplace Collaborator D	Spring Break Cabin-Mate D	**New Basketball Team Player D … has special needs**	Cooking Class Partner D (control)
Material Set 3	Freshman Roommate A	New 2nd-Grade Student A	Workplace Collaborator A	Spring Break Cabin-Mate A	New Basketball Team Player A	Cooking Class Partner A
	**Freshman Roommate B … is blind**	New 2nd-Grade Student B (control)	Workplace Collaborator B	Spring Break Cabin-Mate B	New Basketball Team Player B	Cooking Class Partner B
	Freshman Roommate C	New 2nd-Grade Student C	**Workplace Collaborator C … has special needs**	Spring Break Cabin-Mate C (control)	New Basketball Team Player C	Cooking Class Partner C
	Freshman Roommate D	New 2nd-Grade Student D	Workplace Collaborator D	Spring Break Cabin-Mate D	**New Basketball Team Player D … has a disability**	Cooking Class Partner D (control)
Material Set 4	Freshman Roommate A	New 2nd-Grade Student A	Workplace Collaborator A	Spring Break Cabin-Mate A	New Basketball Team Player A	Cooking Class Partner A
	Freshman Roommate B (control)	**New 2nd-Grade Student B … has special needs**	Workplace Collaborator B	Spring Break Cabin-Mate B	New Basketball Team Player B	Cooking Class Partner B
	Freshman Roommate C	New 2nd-Grade Student C	Workplace Collaborator C (control)	**Spring Break Cabin-Mate C … has a disability**	New Basketball Team Player C	Cooking Class Partner C
	Freshman Roommate D	New 2nd-Grade Student D	Workplace Collaborator D	Spring Break Cabin-Mate D	New Basketball Team Player D (control)	**Cooking Class Partner D … is deaf**
Material Set 5	Freshman Roommate A	New 2nd-Grade Student A	Workplace Collaborator A	Spring Break Cabin-Mate A	New Basketball Team Player A	Cooking Class Partner A
	Freshman Roommate B (control)	**New 2nd-Grade Student B … has a disability**	Workplace Collaborator B	Spring Break Cabin-Mate B	New Basketball Team Player B	Cooking Class Partner B
	Freshman Roommate C	New 2nd-Grade Student C	Workplace Collaborator C (control)	**Spring Break Cabin-Mate C … has ADHD**	New Basketball Team Player C	Cooking Class Partner C
	Freshman Roommate D	New 2nd-Grade Student D	Workplace Collaborator D	Spring Break Cabin-Mate D	New Basketball Team Player D (control)	**Cooking Class Partner D … has special needs**
Material Set 6	Freshman Roommate A	New 2nd-Grade Student A	Workplace Collaborator A	Spring Break Cabin-Mate A	New Basketball Team Player A	Cooking Class Partner A
	Freshman Roommate B (control)	**New 2nd-Grade Student B … has Down syndrome**	Workplace Collaborator B	Spring Break Cabin-Mate B	New Basketball Team Player B	Cooking Class Partner B
	Freshman Roommate C	New 2nd-Grade Student C	Workplace Collaborator C (control)	**Spring Break Cabin-Mate C … has special needs**	New Basketball Team Player C	Cooking Class Partner C
	Freshman Roommate D	New 2nd-Grade Student D	Workplace Collaborator D	Spring Break Cabin-Mate D	New Basketball Team Player D (control)	**Cooking Class Partner D … has a disability**
